# Illustrations of the Effects of Poisons

**Published:** 1834-10-01

**Authors:** 


*Illustrations of the Effects of Poisons.* By GEORGE LEITH ROUPELL, M.D. *The Plates from original Drawings by* ANDREW MELVILLE M'WHINNIE, M.R.C.S. Part II.—London, 1834. 4to.

THIS part, like the former one, contains four cases of poisoning, and four plates representing the post-mortem appearances. The first case is one of poisoning by concentrated sulphuric acid.

"Benjamin Morby, aged two years and a half, drank some fluid which he found in a stone bottle, thinking it to be ginger-beer. It proved, however, to be strong sulphuric acid, left there inadvertently. Immediately after swallowing the poison he fell on the floor; when raised, his tongue was swollen and protruded from his mouth, and he complained of acute pain at the epigastrium. Medical assistance was quickly procured. The carbonate of magnesia was given, which occasioned active effervescence in the mouth. Castile soap in solution was afterwards administered; vomiting then came on, and acid matters, partly composed of food, were rejected from the stomach, followed by a large quantity of black grumous blood. Leeches were applied to the epigastrium. No reaction followed the depression which resulted from the injury to the stomach and œsophagus. The skin was cold. The pulse scarcely perceptible. The little boy remained perfectly sensible and in a state of comparative tranquillity until his death, which took place within twelve hours after the accident.

"The external marks, resulting from the action of the acid, which were discovered by examination after death, consisted in vesication and discoloration of the lips, and in a hard and brawny patch on the left cheek, about the size of a half-crown, and of the colour of parchment.

"The tongue and inner part of the mouth were of a dirty grey colour."

The appearances on dissection are described by Dr. Roupell as follows:

"Plate V. represents the stomach, part of the œsophagus and duodenum, of the little boy poisoned by concentrated sulphuric acid, laid open anteriorly. The inner lining of the œsophagus was puckered, dry, hardened, and brittle; it was readily detached from the parts beneath, and came off in small scale-like portions.

"The coats of the stomach were thin, and allowed the contents to be seen through them. When opened, the whole of the mucous membrane was of a dark colour, apparently stained by a bloody fluid, four ounces of which were contained in the stomach.

"The large end was in no respect altered in texture, but the whole circumference of the smaller end, about midway between the œsophagus and pylorus, was black, irregular, rough, and thickened. The change which had taken place here was the destruction of the mucous membrane and the effusion of blood from the injured portion, some of which had coagulated and adhered to the part acted on by the acid.

"There was no perforation of the coats of the stomach in this instance, nor was there any inflammation of the peritonæum. Life was apparently destroyed by sympathy of the brain with the injury to the stomach and œsophagus."

The next Plate "represents the stomach, part of the œsophagus and duodenum, of a dog poisoned with oxalic acid, opened in the same manner as in the former instances. The hour-glass contraction was manifest.

"The œsophagus was natural in appearance.

"The stomach contained about four ounces of a dark coloured, thick, tenacious fluid, at the larger end, which had stained the mucous surface. The coats were generally pulpy, softened, and had a white transparent appearance, but no perforation had taken place.

"The duodenum was red, and presented dark coloured lines corresponding with the projecting rugæ, which had the appearance of being charred.

"The black matter contained in the stomach was in all probability effused blood, altered by the acid; for healthy mucus, as well as a portion of sound intestine, left in a strong solution of the acid, underwent no change of colour; the latter, however, became gelatinous and pulpy."

The dose of oxalic acid had been two scruples, and death ensued in half an hour. Christison remarks, when speaking of its effects on the human subject, that "among the fatal cases the smallest dose has been half an ounce; but there can be no doubt that less would be sufficient to cause death." (*Treatise on Poisons*, p. 146.)

We next come to an

"*Experiment to shew the effect of a large dose of corrosive sublimate.* A drachm of corrosive sublimate was introduced, as in the last experiment, into the stomach of a dog, and the œsophagus tied.

"The animal exhibited little outward sign of pain, was greatly depressed, but remained tranquil. It was alive between four and five hours after the administration of the sublimate, but was found dead when seen afterwards."

The plate annexed to this case represents the appearances on dissection.

"The stomach externally was highly vascular: it contained several ounces of a thin, dark coloured fluid. The œsophagus was natural. The whole of the inner lining of the stomach was of a leaden hue; this was the mucous membrane, which appeared to be universally in a state of slough, but it was in no part detached. The duodenum, at its commencement, presented a mixed appearance, partly red, partly of a lead colour, as if the irritant and corrosive effects of the poison were here blended; the mucous membrane was thickened, and had a roughened aspect."

"The whole of the small intestines were inflamed, a thick white mucus being generally effused upon their mucous membrane.

"The quantity of corrosive sublimate administered in this case was larger than could probably ever be swallowed, from its nauseous taste and irritant action. The appearances produced by this poison in ordinary cases more nearly resemble the effect here produced on the duodenum."

Dr. Roupell, in supposing that a drachm of corrosive sublimate cannot be swallowed, speaks without book, *i. e.* without Christison; for, at p. 297 of his treatise, we find it stated that "in Devergie's case, the patient, a female, swallowed three drachms of corrosive sublimate in solution."

And in another case, which Dr. Christison has taken from our honoured predecessor, the London Medical and Physical Journal, "the patient, a stout young girl, swallowed, soon after supper, a drachm of corrosive sublimate dissolved in beer." (*Treatise on Poisons*, p. 301.)

The last Plate shows the effect of alcohol on the mucous membrane.

An ounce of rectified spirit, mixed with an ounce of water, was introduced into the stomach of a large dog. The animal soon recovered; it was killed the next day by a blow on the head, and on examination intense congestion was found in the stomach; it would seem too, that extravasation had taken place, as "the stomach contained an ounce of a thin bloody fluid mixed with frothy mucus."

This Part is fully equal to the preceding one; the drawings are admirable, and the colouring, though rich and gorgeous, does not "o'erstep the modesty of nature."

